# Alien hand syndrome as the initial presentation of posterior cerebral artery infarction: a case report

**DOI:** 10.1186/s12883-025-04587-6

**Published:** 2026-01-16

**Authors:** Fatima Alabandi, Zahra Gaw

**Affiliations:** 1 King Fahad Specialist Hospital, Dammam, Saudi Arabia; 2 Dammam Medical Centre, Damma, Saudi Arabia

**Keywords:** Alien hand syndrome (AHS), Posterior cerebral artery (PCA), Supplementary motor area (SMA)

## Abstract

**Background:**

Alien hand syndrome (AHS) is a rare neurological disorder characterized by involuntary, purposeful limb movements without voluntary control. While traditionally associated with frontal or callosal brain lesions, posterior cerebral artery (PCA) involvement remains sparsely reported, making such cases valuable for expanding understanding of the posterior variant of AHS.

**Case Presentation:**

We report a 79-year-old male who presented with a witnessed generalized tonic–clonic seizure followed by acute right-sided weakness. Neurological examination revealed right upper limb movements, described by the patient as disconnected and involuntary. MRI showed an acute ischaemic infarct in the territory of the left posterior cerebral artery (PCA), involving the medial temporal, occipital, and posterior thalamic regions, which is an uncommon aetiology of AHS. The involuntary movements resolved spontaneously within two days without specific pharmacologic therapy.

**Discussion:**

This case is noteworthy for its unique lesion distribution, initial presentation with seizure, and rapid resolution of symptoms. To our knowledge, few reports have described this precise anatomical pattern with transient symptoms. Additionally, the initial presentation with a generalized tonic–clonic seizure preceding alien-hand manifestations represents an uncommon clinical course.

**Conclusion:**

This case underscores the importance of recognizing AHS as a possible early manifestation of posterior circulation stroke, enabling prompt imaging and intervention.

## Introduction

Alien hand syndrome (AHS) is characterized by involuntary, uncontrollable, yet purposeful movements that result from a higher-order motor disorder that is not linked to a specific movement disorder [1].

The first reported case of alien hand syndrome was in 1908 by Kurt Goldstein [2]. However, it was not until 1972 that the phenomenon was more clearly described [3]. Brion and Jedynak coined the term “le signe de la main étrangère” (the sign of the foreign hand) [3], defining it as the denial of hand ownership and the inability to transfer functions between the brain's hemispheres[4]. This definition emerged from their observations of patients with corpus callosum tumours [4] who were unable to recognize their own left arms without visual input [3].

This condition can be caused by midline tumours, callosotomy, and, less commonly, stroke [5]. However, most of the reported cases involved lesions of the corpus callosum or frontal lobe, either alone or in combination [3].

In most documented cases of alien hand syndrome (AHS) related to strokes in the literature, the responsible cerebral lesions are primarily found in the frontal lobe and often involve the anterior corpus callosum [6]. Posterior or sensory variants of AHS are rarely reported and have been previously linked to lesions of the parietal or thalamic regions [1, 6, 7]. Our case expands on these findings by illustrating that combined occipital, medial temporal, and posterior thalamic infarction can also produce similar phenomena, likely through disruption of visuospatial and proprioceptive integration networks. While the transient course precludes definitive mechanistic conclusions, the clinical and radiological correlation underscores the heterogeneity of posterior AHS presentations. The novelty of this case lies in this specific lesion pattern, the initial seizure presentation, and the rapid recovery of alien hand symptoms within two days.

## Case

A 79-year-old man with no prior medical history was brought to a private hospital after a witnessed generalized tonic–clonic seizure lasting 5–10 min, followed by postictal drowsiness for 30 min and right-sided weakness. Upon transfer to our facility, he was alert and oriented to time, person, and place.

Neurological examination revealed normal extraocular movements, and visual field assessment confirmed no homonymous hemianopia. He had moderate dysarthria, flattening of the right nasolabial fold, and mild facial sensory loss in the distribution of V1–V3. Right upper limb power was 3/5, compared with 5/5 in the other limbs. Finger-to-nose testing was intact bilaterally.

During observation, the patient exhibited involuntary but seemingly purposeful movements of the right upper limb, occasionally striking his own body or face, without conscious control. The patient repeatedly described the limb as “moving on its own”. He reported being unable to restrain it when the abnormal movements occurred. These movements were witnessed by the neurology team and gradually resolved over two days without specific pharmacologic intervention.

The initial brain CT scan revealed atrophic brain parenchyma and evidence of chronic microangiopathy but no haemorrhage, ischaemic events, or brain mass. The laboratory workup was largely unremarkable. Additionally, the ECG showed a normal sinus rhythm. The patient was loaded with aspirin and admitted for further stroke work-up and management, including brain magnetic resonance imaging (MRI).

**Fig. 1:**
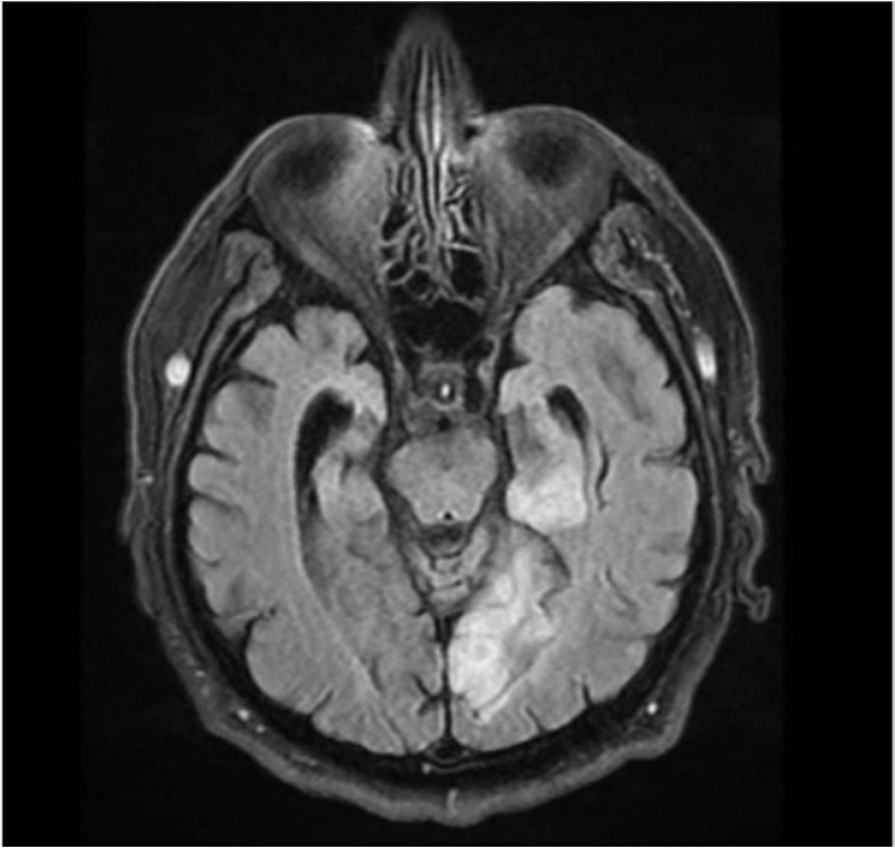
Left meso-temporal hyperintense signal in T2-weighted and FLAIR sequences

**Fig. 2:**
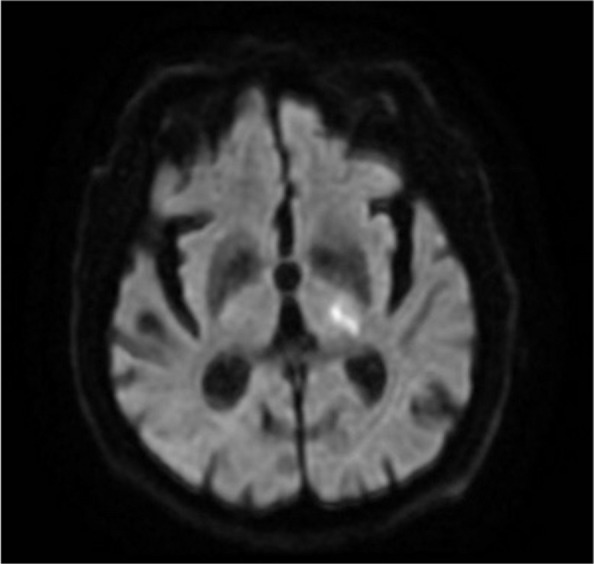
Restricted diffusion in the left thalamus on DWI MRI

**Fig. 3:**
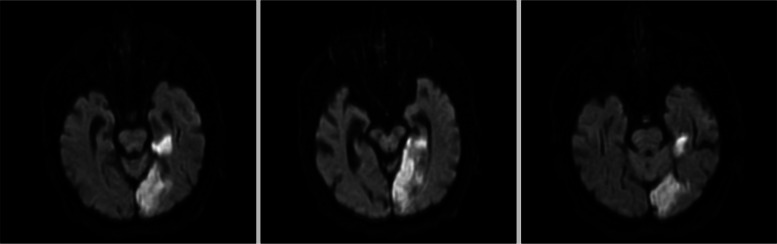
Restricted diffusion in the left occipital lobe on DWI MRI

MRI of the brain was performed within 48 h, revealing significant left medial temporal, occipital, and posterior thalamic restricted diffusion, along with hyperintense signals on FLAIR imaging (Figs. 1, 2 and 3). These findings are consistent with acute ischaemic stroke in the territory of the left posterior cerebral artery.

## Discussion

The key features of AHS include the feeling of foreignness of the limb; failure to recognize ownership of the limb when visual cues are removed; or a sensation that the limb acts with a will of its own [6]. The affected limb can be either the arm or the leg [3].

AHS is a rare but documented manifestation of stroke [5]. In one reported case, involuntary abnormal limb movement was the earliest symptom observed by the patient, who was later found to have developed a right thalamic infarction [7].

There are three main variants of alien hand syndrome (AHS) based on clinical and anatomical perspectives [2, 6]: frontal and callosal AHS, which involves motor circuits, and a posterior variant, known as sensory AHS, which is observed following posterior cerebral artery infarction [6]. The latter could be attributed to the cause of AHS in our patient.

The frontal variant arises from lesions in the supplementary motor area (SMA), cingulate cortex, dominant medial prefrontal cortex, or corpus callosum. It typically presents with groping (where the hand appears to be constantly searching for nearby objects), grasping, or compulsive manipulation of tools [8].

The callosal variant usually involves the nondominant hand and primarily results from callosal damage. It often involves intermanual conflict, characterized by opposing purposeful movements of the patient's hands. In this variant, it is common to observe the hands “fighting” against each other when trying to complete a goal-directed activity [8].

The posterior variant, as encountered in this case, has recently been recognized as a consequence of damage to the thalamus, posterolateral parietal lobe, or occipital lobe [8]. Patients with this variant may unintentionally withdraw the affected hand from environmental contact or stimuli (an avoidance response) or experience uncoordinated hand movements or involuntary levitation [8].

Nonetheless, in a case report of a female with callosal haemorrhage extending to the medial frontal lobe, she was noted to exhibit both intermanual conflict and compulsive grasping movements, which shows that patients may simultaneously demonstrate features of different variants [8].

In terms of pathophysiology, functional brain MRI has been used to study brain activity in patients with alien hand syndrome (AHS) [5]. In normal individuals, the initiation of motor activity activates multiple extensive neural networks [5]. In contrast, patients with AHS show only isolated activation of the contralateral primary motor cortex [5]. In typical frontal/callosal cases, where motor pathways are abnormally activated, callosal pathology is presumed to disrupt the normal transfer of information to the opposite hemisphere [1]. Additionally, spontaneous movements occurring without the patient’s awareness or intention in cases of parietal cortex pathology are thought to arise from a combination of loss of proprioceptive feedback and hemineglect that together results in a lack of awareness of movements [5].

The features associated with AHS differ according to the variant. Posterior AHS has been noted in association with optic ataxia, hemianaesthesia, hemianopia, visuospatial neglect, and optic ataxia, which are typically the consequence of PCA infarction [1]. Therefore, it has been suggested that this syndrome be categorized as “sensory” or “posterior” to distinguish it from the more commonly recognized motor or anterior variant [7].

In our case, the patient’s involuntary right upper limb movements were consistent with the posterior (sensory) variant of AHS, in which disrupted proprioceptive feedback leads to uncoordinated or self-directed actions. Additionally, his brain MRI demonstrated infarction in the posterior thalamus, medial temporal, and occipital cortices, which is an unusual combination not frequently reported in the literature. The posterior thalamus acts as a sensory relay integrating proprioceptive and visual information, while occipitotemporal regions contribute to visuospatial processing and awareness. Disruption across these interconnected regions likely produced the transient disownership and involuntary movements characteristic of posterior AHS.

Prior reports [1, 6, 7] describe sensory AHS following parietal or thalamic strokes, often accompanied by hemianopia or sensory loss. In contrast, our patient exhibited minimal sensory or visual field deficits, suggesting partial sparing of these networks and possibly explaining the rapid recovery. Moreover, the rapid resolution within two days sets this case apart from previously reported posterior AHS cases, where symptoms often persist for weeks or months [1, 6, 7].

The prognosis of patients with alien hand syndrome (AHS) varies in the literature. Typically, symptoms decrease within one week but can persist for more than a year [6]. A literature review of 18 cases of stroke-related AHS suggested that the prognosis is better for AHS following right brain stroke than for left brain stroke [3].

In terms of management, various rehabilitation techniques have been suggested, such as mirror box therapy, the use of objects such as balls to distract the affected limb, and the visualization of the requested tasks [3]. Trials involving clonazepam, carbamazepine, and botulinum toxin A injections into the affected arm have been reported, but their efficacy has not yet been thoroughly studied [3].

## Conclusion

Alien limb phenomena were previously linked to neurodegenerative disorders; however, our case, along with previous literature, demonstrates that alien limb phenomena can occur acutely due to ischaemic lesions, reinforcing the need to suspect stroke when such symptoms arise within the therapeutic window.

## Data Availability

All data supporting the conclusions of this article are included within the manuscript.
